# A comparative evaluation of canal transportation and centering ability of 3 different rotary file systems in oval canals: An *in vitro* CBCT study

**DOI:** 10.6026/973206300212853

**Published:** 2025-08-31

**Authors:** Shreya Saurabh, Deepak Kurup, Shazia Mahreen, Ramsha Rizwan, Garima Sinha, Sourav Tikadar

**Affiliations:** 1 Department of Conservative Dentistry & Endodontics, Hazaribag College of Dental Sciences & Hospital, Harazibagh, Jharkhand, India

**Keywords:** CBCT, canal transportation, centering ability, rotary NiTi files, oval canals

## Abstract

Achieving proper canal shaping in oval root canals is a significant challenge in endodontics, as anatomical variations often lead to procedural errors such as canal transportation and poor centering. Sixty extracted mandibular premolars with standardized root length and confirmed oval canals were randomly assigned into three groups (n=20 each) and instrumented according to manufacturer's protocols. CBCT scans at 3 mm, 6 mm and 9 mm from the apex were analyzed for centering ratio and canal transportation, with results compared using one-way ANOVA (p<0.05). Hyflex CM demonstrated the best centering ability at 9 mm mesiodistally, Jizai showed the least transportation at the same level and One Curve performed better at 3 mm buccolingually. Thus, we show that rotary file systems differ in their ability to maintain canal anatomy and instrument selection plays a crucial role in achieving optimal root canal shaping.

## Background:

Important phases in endodontic treatment include properly cleaning and shaping the root canal system. During this stage, the canals are shaped using either hand-operated or engine-driven tools, which is generally acknowledged as an essential step in the successful completion of a root canal procedure [1]. Maintain a continuous, tapered shape while preserving the apical foramen's original configuration and location is a crucial objective during instrumentation. However, this task is made more difficult by root canal curvatures [2]. Canal transportation is one of the difficulties brought on by these curvatures. As instruments tend to return to their natural linear shape during the shaping process, canal transportation-defined by the Glossary of Endodontic Terms as "the removal of canal wall structure on the outer curve in the apical half of the canal"-occurs. On the other hand, centering ability describes the instrument's ability to stay centred inside the canal, which is essential for appropriate enlargement without excessive weakening of the root structure [3]. Long oval canal is common in the apical 5 mm in human teeth. Many long and narrow oval canals would be impossible to instrument completely without perforating or significantly weakening the roots [4]. Research has indicated that the clinical outcomes of rotary file systems are significantly influenced by the design and manufacturing process of NiTi instruments [5]. However, canal curvature continues to be a difficult risk factor and during endodontic treatment, procedural errors like ledge formation, canal straightening and zipping and canal transportation remain major concerns [2, 6]. Because shaping directly affects subsequent steps like irrigation and obturation, it is critical for overall treatment success [7]. Inadequate instrument flexibility and poor centering ability frequently result in canal transportation. The advent of rotary nickel-titanium (NiTi) tools in contemporary endodontics has made it possible to prepare canals more effectively, thereby reducing the risk of procedural errors. A crucial stage in efficient canal shaping is the creation of a glide path-a smooth radicular tunnel extending from the canal orifice to the physiological terminus (foraminal constriction). This glide path must be created before using rotary or reciprocating systems to preserve natural anatomy. Although the glide path can be created with both hand and rotary instruments, hand files are often considered time-consuming, especially when dealing with severe curvatures or obliterated canals [8].

ProGlider rotary glide path files have been reported to reduce canal transportation and deviation compared with manual files [9]. The One Curve Endo DNA (MicroMega) single-file system was released after further advancements in NiTi technology. This single-use rotary file, made of heat-treated NiTi alloy, enables full-length canal shaping up to the apex using a single tool. Its patented variable cross-section along the blade enhances centering ability in the apical third and improves debris clearance in the middle and coronal thirds. According to the manufacturer, this file demonstrates 2.4 times greater cyclic fatigue resistance than its predecessor, One Shape and reduces root canal preparation time by 33% compared to reciprocating single-file systems. Another innovation in NiTi instruments is the HyFlex CM file system (Coltene Whaledent AG, Altstatten, Switzerland), manufactured using a thermomechanical process of CM-wire. This technology reduces ledging, transportation and perforation by providing superior flexibility and shape memory control. HyFlex CM instruments closely follow the canal anatomy and maintain canal integrity during continuous rotation. The Jizai file system represents advancement in rotary NiTi instruments. Made from a special heat-treated NiTi alloy, its asymmetrical, off-centered quasi-rectangular cross-section provides reduced screw-in forces and greater space for debris removal [10]. Therefore, it is of interest to compare the centering ability and canal transportation of HyFlex CM, One Curve and Jizai rotary file systems in oval-shaped root canals using CBCT analysis.

## Material and Methodology:

## Tooth selection:

Sixty extracted human mandibular premolars, each possessing a single, oval-shaped root canal, were chosen for this study.

## Standardization of samples:

Each tooth was decoronated to achieve a uniform root length of 12 mm.

## Access cavity preparation:

Standard endodontic access cavities were created using high-speed diamond burs with water cooling. Patency was verified using#10 K-file and the working length was established. A glide path was then prepared to the full working length with the Dentsply ProGlider file.

## Grouping of samples:

The sixty standardized teeth were randomly divided into three experimental groups, with 20 teeth in each group:

[1] Group I: Jizai file system

[2] Group II: One Curve file system

[3] Group III: Hyflex CM file system

## Root canal preparation:

In accordance with the manufacturer's suggested instrumentation protocol, each group had a root canal prepared using the appropriate file system.

## Method of assessment:

All samples underwent pre- and post-instrumentation cone beam computed tomography (CBCT) scans to evaluate canal morphology and the modifications brought about by the mechanical preparation.

## Statistical analysis:

For each of the three experimental groups (Type 1, Type 2 and Type 3), the mean and standard deviation of the canal transportation and centring ratio values were calculated at three different measurement levels: 3 mm, 6 mm and 9 mm from the apex. SPSS software (version 19.0.0.0; IBM Corp., Armonk, NY) was used to analyse the data. One-way analysis of variance (ANOVA) was used to determine whether the data was normal. For every group, descriptive statistics such as means and standard deviations (SD) were computed. To ascertain statistical significance, ANOVA was used for both intragroup and intergroup comparisons.

## Results:

Tables 1-4 (see PDF) provide a summary of the findings from the transportation and canal centring analyses. The results of the ANOVA analysis showed statistical significance with a P-value of 0.000 (P < 0.05). At 3 mm and 6 mm, there were no statistically significant differences in the three groups' centering ability (P < 0.001). Significant variations were observed at 9 mm in the mesiodistal plane, though, with Group 3 (Hyflex CM) outperforming the other two in terms of centering ability. Furthermore, Group 3 (Hyflex CM) displayed the highest degree of canal transportation at 9 mm in the mesiodistal plane, while Group 1 (Jizai) displayed the least amount. At every measurement level, there were no statistically significant differences in centering ability between Groups 1 and 3 in the intragroup comparisons. Group 2 (One Curve), on the other hand, demonstrated noticeably superior centering ability in the buccolingual plane at 3 mm. In comparison to the other groups, Group 3 showed the least amount of canal transportation (3 mm), indicating that it was effective in maintaining the original canal structure.

## Discussion:

This study used cone beam computed tomography (CBCT) to assess the centering ability and canal transportation of the Jizai, One Curve and Hyflex CM rotary file systems in oval-shaped root canals of mandibular premolars. The evaluation of the centering ratio and canal transportation offers crucial information about the quality of root canal preparation accomplished by various tools and methods [1, 2]. Root canal instrumentation aims to enlarge the canal while maintaining its original anatomy and minimizing procedural errors such as canal transportation, zipping, ledging, or perforations [3]. The distal roots of lower molars, upper and lower premolars, lower incisors and canines frequently have oval or flat canal morphology [4]. According to Wu *et al.*, up to 25% of root canals have an oval or flat shape; in some root groups, this percentage can even surpass 50% [5]. Oval-shaped canals are particularly challenging because conventional rotary instruments tend to prepare the central region of the canal while leaving untouched areas in the buccolingual extensions, which may harbor debris and microorganisms [6, 7]. Therefore, assessing the shaping ability of new instrument systems in such anatomies is essential. Three cross-sectional levels were chosen in this study to represent the apical, middle and coronal thirds of the root canals: 3 mm, 6 mm and 9 mm. In order to capture the variation in canal shape at various root levels, these measurement points were selected [8]. CBCT has been validated as a reliable tool for assessing canal transportation and centering ratio, offering high-resolution 3D reconstructions without destructive sectioning of samples [9]. Group 3 (Hyflex CM) showed the least amount of canal transportation at 3 mm in terms of centering ability and canal transportation, indicating that it is effective in maintaining the canal's original structure at this level. This finding corroborates earlier studies demonstrating that controlled memory (CM) instruments, due to their superior flexibility and fatigue resistance, adapt more closely to canal curvature and reduce transportation in the apical third [10, 11]. However, in intergroup comparisons, Group 1 (Jizai) showed the least amount of canal transportation at the same level, while Group 3 showed the most at 9 mm in the mesiodistal plane. This variability highlighted that different rotary systems may perform differently across canal levels depending on their design, metallurgy and taper [12]. During instrumentation of the root canal, it is important to develop a continuously tapered form and to maintain the original shape and position of the apical foramen [13]. These results align with previous investigations showing that no single rotary system is universally superior across all canal levels and that instrument choice should be based on anatomical considerations [14]. Overall, the findings of this study emphasize that maintaining canal curvature with minimal transportation and achieving good centering ability are essential goals in root canal preparation [15].

## Conclusion:

The three rotary systems showed different shaping abilities at various canal levels. No single file system proved universally superior, as each demonstrate distinct strengths in preserving canal anatomy. Therefore, instrument selection should be based on canal morphology and clinical requirements.
